# Efficacy and Tolerability of a Novel Cosmetic and Over‐the‐Counter Facial Acne Regimen Versus a Prescription Treatment

**DOI:** 10.1111/jocd.16568

**Published:** 2024-09-20

**Authors:** Priscilla Huang, Olivia Supan, Cecilia L. Pak, Rahul C. Mehta, Elizabeth T. Makino

**Affiliations:** ^1^ Allergan Aesthetics, an AbbVie Company Irvine California USA; ^2^ SGS Stephens Inc. Richardson Texas USA

**Keywords:** acne vulgaris, erythema, post‐inflammatory hyperpigmentation, salicylic acid

## Abstract

**Background:**

The SkinMedica Acne Treatment Platform (SM Regimen) was formulated to treat acne without overdrying the skin. We evaluated efficacy and tolerability of the SM Regimen (including a novel 1% salicylic acid Acne Clarifying Cleanser and 2% salicylic acid Acne Treatment Lotion) versus a prescription formulation (Rx Regimen; including adapalene 0.1%/benzoyl peroxide 2.5%) in a diverse population of adults with mild to moderate facial acne.

**Methods:**

This single‐center, double‐blind, randomized study enrolled adults (18–45 years) with Fitzpatrick skin types (FST) I–VI. SM Regimen or Rx Regimen was applied topically to the entire face for 12 weeks. Assessments were conducted at 24 and 48 h and 4, 8, and 12 weeks.

**Results:**

Subjects (SM Regimen, *n* = 31; Rx Regimen, *n* = 23) were primarily female (90.7%) with mean age of 28.6 years; 53.8% had FST IV–VI. Efficacy was comparable between regimens. The SM regimen resulted in significant improvements versus baseline in mean Investigator's Global Assessment of acne severity from 48 h through week 12 (*p* ≤ 0.001), as well as significant and sustained improvements from baseline in total acne lesion count, global postinflammatory hyperpigmentation/postinflammatory erythema, and oiliness. The SM Regimen was well tolerated at all time points, with mean scores below mild for all parameters; the Rx Regimen caused significantly more tightness/dry feeling at week 4 versus SM Regimen (*p* = 0.008). Subjects (> 96%) reported high satisfaction with the SM Regimen at all time points.

**Conclusions:**

The SM Regimen reduced acne severity and skin oiliness, evening out skin tone without overdrying or irritating the skin.

## 1 Introduction

Acne vulgaris development is multifactorial, resulting in part from blocked follicles, keratinocyte hyperproliferation, increased sebum production and excretion, the presence of *Cutibacterium acnes* within the follicle, and inflammatory mediators released from nearby skin cells [1, 2]. Acne lesions and other sequelae, including postinflammatory erythema (PIE), postinflammatory hyperpigmentation (PIH), and scarring, can negatively affect psychosocial well‐being and quality of life [3, 4, 5, 6].

Adult acne, occurring after 25 years of age, typically presents as mild to moderate facial inflammatory papulopustular lesions [7], and has a higher prevalence in women (12%) versus men (3%) [8]. Inflammatory lesions may result from chronic stimulation of the innate immune system by resistant strains of *C. acnes* [3]. PIH and scarring are frequent among women [8] and can be more prevalent and prominent in adults with Fitzpatrick skin types (FST) IV, V, and VI who may require long‐term therapy for control [9, 10].

Current treatments for mild to moderate acne include topical formulations containing benzoyl peroxide (BP; antimicrobial), adapalene (retinoid), salicylic acid and azelaic acid (keratolytic), sulfur (keratolytic and bacteriostatic), and antibiotics [11]. Salicylic acid, found in both over‐the‐counter and prescription topical agents, is effective against inflammatory and noninflammatory acne lesions. Some local skin irritation is possible at higher, prescription doses of salicylic acid (> 2%) [2, 12]. Adapalene 0.1% combined with BP 2.5% (Epiduo; Galderma, Lausanne, Switzerland), a widely used, prescription topical combination, has demonstrated efficacy for the treatment of moderate to severe acne [13]. Although adapalene 0.1%/BP 2.5% was well tolerated in clinical studies, potential adverse events of dryness and scaling may limit its use, particularly for individuals with dry, sensitive, or aging skin [11, 14]. There is an unmet need for treatments suitable for mild to moderate acne in all skin types and gentle enough for adults with aging skin, who may require extended treatment.

The SkinMedica Acne Treatment Platform is a topical combination regimen consisting of a cosmetic Acne Clarifying Cleanser and over‐the‐counter Acne Treatment Lotion (SM Regimen) that acts multimodally to target pathways contributing to acne formation. The Acne Clarifying Cleanser is a blend of 1% salicylic acid with bakuchiol (antioxidant, anti‐inflammatory, antibacterial), bisabolol (anti‐inflammatory), aloe and olive leaf (soothing), and licorice root extract (antioxidant). The Acne Treatment Lotion contains a blend of encapsulated 2% salicylic acid and bakuchiol, niacinamide (antioxidant, anti‐inflammatory), and *Centella asiatica* extract (anti‐inflammatory, antioxidant) for exfoliating acne‐causing dead skin, oil, and dirt without overdrying and irritating the skin; and a synergistic complex of manuka, black cumin, magnolia, and chaulmoogra for sebum control, prevention of sebum oxidation, and antimicrobial effects [15, 16, 17, 18]. The oxidation of lipid components of sebum, such as squalene, can stimulate keratinocyte proliferation and the release of inflammatory mediators [19]. By modulating sebum oxidation, the SM Regimen may help address this mechanism of acne pathogenesis.

This study evaluated the efficacy and tolerability of the SM Regimen compared with a topical prescription formulation (adapalene 0.1%/BP 2.5%; Rx Regimen) over 12 weeks in a diverse population of adults with acne, including those with PIH/PIE.

## 2 Materials and Methods

### 2.1 Study Design

This single‐center, double‐blind, randomized clinical study was conducted from June 13, 2022 to March 22, 2023. Healthy males and females, 18–45 years of age with FST I–VI, who had mild to moderate severity of facial acne (scores of 2–3 on the US Food and Drug Administration [FDA] Investigator's Global Assessment [IGA] of acne scale; 0 = clear skin, 4 = severe) were enrolled. Eligible subjects had ≥ 2 inflammatory lesions and 10–100 noninflammatory lesions on the face, ≥ 1 isolated inflammatory lesion for target grading, and no facial treatments in the past 6 months, and were willing to withhold all facial treatments for improving the appearance or firmness of facial skin throughout the study. The target enrollment was 65 subjects, with at least 50 subjects expected to complete the trial (30 subjects in the SM Regimen group and 20 subjects in the Rx Regimen group). In the SM Regimen group, ≥ 15 subjects were required to have FST I–III, with the remainder having FST IV–VI, and ≥ 15 subjects had to have mild PIH/PIE acne marks (score ≥ 1 according to a modified Griffith's 10‐point scale [0 = none, 9 = severe]). In the Rx Regimen group, ≥ 10 subjects were required to have FST I–III, with the remainder having FST IV–VI, and ≥ 10 subjects had to have mild PIH/PIE acne marks.

Key exclusion criteria were history of skin cancer within the past 5 years or current or prior use of the oral or topical treatments (e.g., oral isotretinoin within 6 months, oral or topical prescription medications for acne within 30 days, any systemic medication considered to affect the course of acne within 30 days [e.g., antibiotics, steroids]; over‐the‐counter retinol‐containing topical or systemic products within 30 days; and other topical over‐the‐counter products within 14 days).

Subjects were randomized to receive the SM Regimen, consisting of Acne Clarifying Cleanser (salicylic acid 1%) and Acne Treatment Lotion (salicylic acid 2%), or the Rx Regimen, consisting of adapalene 0.1% and BP 2.5%. In the SM Regimen group, subjects applied the Acne Clarifying Cleanser and Acne Treatment Lotion on the full face twice daily (am/pm). In the Rx Regimen group, subjects applied adapalene 0.1% and BP 2.5% once daily at night, in accordance with the prescribing information. Both groups used SkinMedica Facial Cleanser (am/pm), SkinMedica Ultra Sheer Moisturizer (am/pm), and SkinMedica Essential Defense Mineral Shield SPF 35 (am only). Study visits occurred at baseline, 24 and 48 h, and 4, 8, and 12 weeks.

The study was approved by an institutional review board (Advarra IRB, Columbia, MD, USA) and conducted in accordance with all applicable guidelines for the protection of human subjects for research as required by 21 Code of Federal Regulations (CFR) 50.25 and the accepted standards for Good Clinical Practice. All subjects provided written informed consent before study enrollment.

### 2.2 Endpoints and Assessments

#### 2.2.1 FDA IGA of Acne Severity

Investigators assessed global acne severity at each visit on a 5‐point scale as clear skin (grade 0), almost clear (grade 1), mild (grade 2), moderate (grade 3), and severe (grade 4).

#### 2.2.2 Acne Lesion Counts

Investigator‐assessed counts of total acne lesions, inflammatory lesions (papules, pustules), and noninflammatory lesions (open and closed comedones) on the face were recorded at each visit. Lesion counts were assessed separately for the forehead, left and right cheeks (including the side of nose), and chin (including the area above the upper lip).

#### 2.2.3 Target Inflammatory Lesion Assessment

Target lesions were papules or pustules emerging within 48 h of baseline and between 0.1 and 0.5 cm in size. Investigator‐assessed clinical grading of 1 or 2 target lesions was performed at baseline and at 24 and 48 h. Lesions were graded for redness (score of 0 [none] to 4 [severe]), elevation (score of 0 [completely flat] to 4 [severely raised/very “swollen”]), and size/diameter (score of 0 [not visible]; 1 [≤ 1‐mm diameter]; 2 [> 1‐ to 2‐mm diameter]; 3 [> 2‐ to 3‐mm diameter]; 4 [> 3‐mm diameter]).

#### 2.2.4 PIH/PIE Assessments

Global PIH/PIE assessment of the entire face was evaluated at each visit using 10‐point modified Griffith's scale as none (score of 0), mild (1–3), moderate (4–6), and severe (7–9). Target PIH/PIE lesions (1 or 2) were graded at baseline and 4, 8, and 12 weeks for darkness (score of 0 [equal to tone/color of surrounding skin, no visible PIH/PIE] to 6 [extremely darker/redder than surrounding skin]) and size (score of 0 [not visible]; 1 [≤ 1‐mm diameter]; 2 [> 1‐ to 2‐mm diameter]; 3 [> 2‐ to 3‐mm diameter]; 4 [> 3‐mm diameter]).

#### 2.2.5 Tactile Oiliness

Investigator‐assessed clinical grading of tactile oiliness was performed at each visit using the following scale: 0 (no oily feel); 1–3 (mild oily feel); 4–6 (moderate oily feel); and 7–9 (strong oily feel).

#### 2.2.6 Subject Self‐Assessment

Subjects completed a sponsor‐provided self‐assessment questionnaire at 24 and 48 h and 4, 8, and 12 weeks to record their experience and level of satisfaction with the test products.

#### 2.2.7 Imaging Procedures

VISIA‐CR imaging was performed at each visit. Subjects were photographed using the VISIA‐CR photo‐station (Canfield Imaging Systems, Fairfield, NJ, USA) with a Canon Mark digital SLR camera (Canon Inc., Tokyo, Japan).

#### 2.2.8 Tolerability Assessments

Tolerability evaluations were performed at each visit. Investigator‐assessed erythema and dryness/scaling were assessed on a 10‐point scale (0 = none, 1–3 = mild, 4–6 = moderate, 7–9 = severe) and edema on a 4‐point scale (0 = none, 1 = mild, 2 = moderate, and 3 = severe). Subject assessments of burning, itching, and tightness/dry feeling were reported on a 4‐point scale (0 = none, 1 = mild, 2 = moderate, and 3 = severe).

### 2.3 Statistical Methods

Target enrollment was based on the number of patients typically enrolled in past studies investigating topical skincare products. Analyses were conducted in the intent‐to‐treat (ITT) population, which included all treated subjects with at least 1 post‐baseline evaluation. Between‐group comparisons and change from baseline were analyzed by Wilcoxon signed rank test. Statistical tests were 2‐sided (*α* = 0.05) and performed using SAS software version 9.4 (SAS Statistical Institute).

## 3 Results

### 3.1 Subjects

A total of 63 subjects were enrolled and 54 subjects were included in the ITT population (SM Regimen, *n* = 31; Rx Regimen, *n* = 23; Table 1). Most subjects were female (90.7%), and there was a balanced proportion of subjects with FST IV–VI (> 50% in both groups). In the SM Regimen versus the Rx Regimen group, respectively, there were numerically lower proportions of White subjects (32.3% vs. 47.8%) and Black or African American subjects (41.9% vs. 47.8%) and a higher proportion of Asian subjects (25.8% vs. 4.3%). A total of 14 subjects discontinued the study (SM Regimen, *n* = 9; Rx Regimen, *n* = 5) due to subject‐requested withdrawal (SM Regimen, *n* = 3; Rx Regimen, *n* = 2), lost to follow‐up (SM Regimen, *n* = 5; Rx Regimen, *n* = 2), did not meet protocol criteria (SM Regimen, *n* = 1; Rx Regimen, *n* = 0), and noncompliance (SM Regimen, *n* = 0; Rx Regimen, *n* = 1).

**TABLE 1:** Subject demographics and baseline characteristics.

	SM Regimen (*n* = 31)	Rx Regimen (*n* = 23)	Total (*N* = 54)
Age, mean (SD) years	28.8 (7.7)	28.3 (6.6)	28.6 (7.2)
Gender, *n* (%)			
Female	27 (87.1)	22 (95.7)	49 (90.7)
Male	4 (12.9)	1 (4.3)	5 (9.3)
Ethnicity, *n* (%)			
Hispanic or Latino	1 (3.2)	3 (13.0)	4 (7.4)
Not Hispanic or Latino	30 (96.8)	20 (87.0)	50 (92.6)
Race, *n* (%)			
Asian	8 (25.8)	1 (4.3)	9 (16.7)
Black/African American	13 (41.9)	11 (47.8)	24 (44.4)
White/Caucasian	10 (32.3)	11 (47.8)	21 (38.9)
Fitzpatrick skin type, *n* (%)			
I–III	14 (45.2)	11 (47.8)	25 (46.3)
IV–VI	17 (54.8)	12 (52.2)	29 (53.7)

### 3.2 Efficacy

#### 3.2.1 IGA of Acne Severity

**FIGURE 1:**
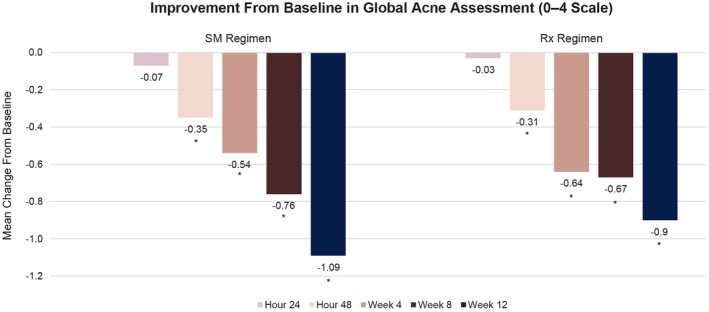
Change from baseline in mean IGA acne severity assessment over time. No statistical significance was observed between treatment groups. IGA, Investigator's Global Assessment. *p ≤ 0.004 versus baseline.

For both treatment groups, significant improvements versus baseline were observed in mean global acne severity scores at each follow‐up visit from 48 h through 12 weeks (*p* ≤ 0.004; Figure 1). No statistically significant differences in mean scores between treatments were observed.

#### 3.2.2 Acne Lesion Counts

**FIGURE 2:**
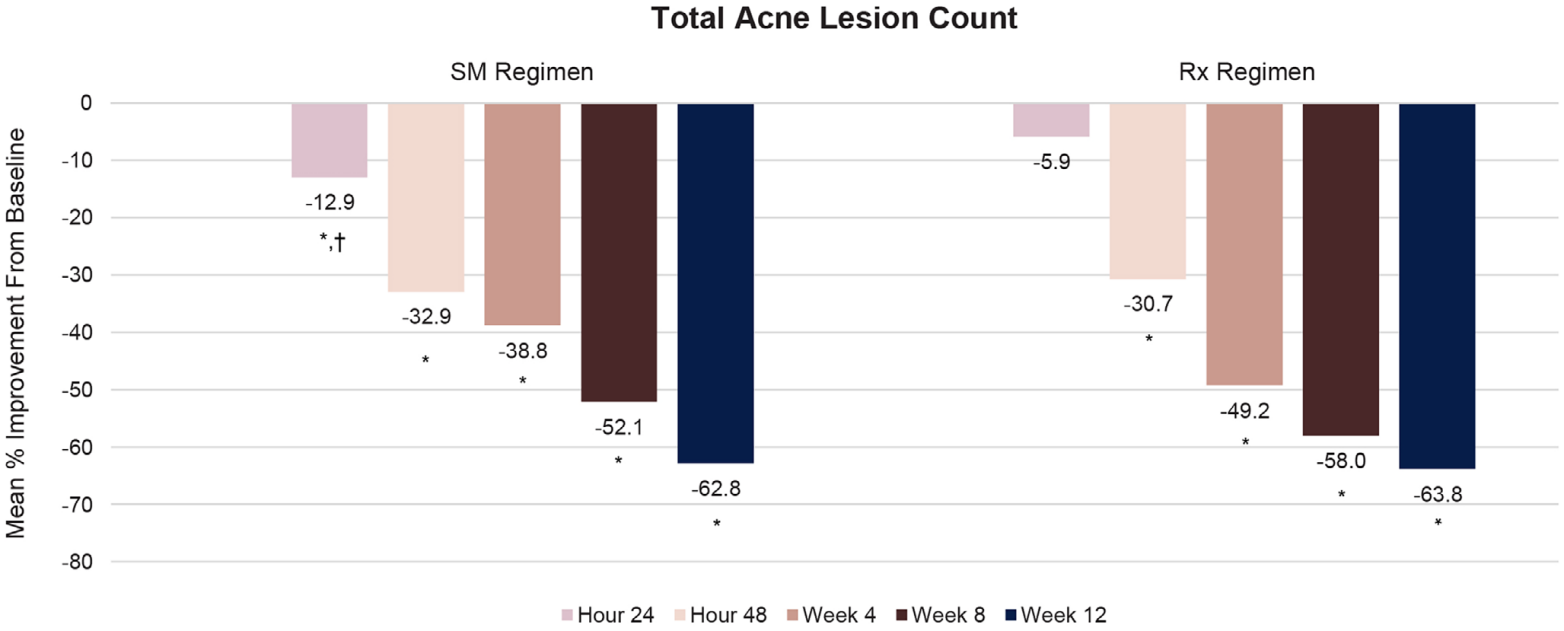
Percent change from baseline in mean total acne lesion count over time. *p < 0.001 versus baseline. ^†^ *p* = 0.021 versus Rx Regimen.

Significant improvements in total acne lesion counts versus baseline were observed at 24 h for the SM Regimen and at 48 h for the Rx Regimen, with significant improvements at all subsequent time points (*p* ≤ 0.001; Figure 2). No significant differences between treatments were observed except at 24 h, when the SM Regimen showed greater improvement from baseline versus the Rx Regimen (*p* = 0.021).

The SM Regimen showed significant improvement in noninflammatory lesion count compared both with baseline (*p* < 0.001) and with the Rx Regimen (*p* = 0.016) at 24 h (Table 2). Significant improvements from baseline were observed for both the SM Regimen and Rx Regimen from 48 h up to 12 weeks (*p* < 0.001).

**TABLE 2:** Mean percentage change from baseline in investigator assessments.

Mean percentage change from baseline	SM Regimen					Rx Regimen				
	24 h	48 h	Week 4	Week 8	Week 12	24 h	48 h	Week 4	Week 8	Week 12
Acne lesion count										
Noninflammatory	−15.3%*^,§^	−37.0%*	−42.8%*	−55.4%*	−66.6%*	−5.0%	−31.1%*	−52.2%*	−62.8%*	−68.3%*
Inflammatory	−3.0%	−15.3%	−21.6%	−37.9%^†^	−45.4%*	−10.2%	−28.6%^†^	−39.2%*^,§^	−41.2%*	−46.4%*
Target inflammatory lesion assessment										
Redness	−16.9%*	−37.3%*	—	—	—	−13.5%^†^	−32.7%*	—	—	—
Elevation	−20.7%*	−45.6%*	—	—	—	−15.7%^†^	−40.4%*	—	—	—
Size/Diameter	−2.7%	−18.3%^†^	—	—	—	−1.7%	−15.3%^‡^	—	—	—
Target PIH/PIE lesion grading										
Darkness	—	—	−25.2%*	−40.2%*	−56.2%*	—	—	−26.8%*	−37.6%*	−55.1%*
Size	—	—	−25.2%*	−46.1%*	−57.9%*	—	—	−20.8%^†^	−31.4%*	−47.2%*

*Note:* Indicates statistically significant improvement versus baseline: *p < 0.001; ^**†**^ *p* < 0.005; ^**‡**^ *p* < 0.05. Indicates statistically significant improvement versus the other group: ^§^ *p* < 0.05.

Abbreviation: PIH/PIE, postinflammatory hyperpigmentation/postinflammatory erythema.

Compared with baseline, significant improvements in mean inflammatory lesion count were observed starting at 8 weeks for the SM Regimen (*p* = 0.003) and at 48 h for the Rx Regimen (*p* = 0.005), with significant improvement versus baseline in both groups up to 12 weeks (Table 2). No statistically significant differences in mean inflammatory lesion count between treatments were observed, except at 4 weeks, when change from baseline was significantly greater for the Rx Regimen versus the SM Regimen (*p* = 0.036).

#### 3.2.3 Target Inflammatory Lesion Assessments

Compared with baseline, both regimens showed significant reductions in redness and elevation of target inflammatory lesions at 24 and 48 h (*p* ≤ 0.004) and in size at 48 h (*p* ≤ 0.008) (Table 2). No statistically significant differences in target inflammatory lesions between groups were observed.

#### 3.2.4 PIH/PIE Assessments

**FIGURE 3:**
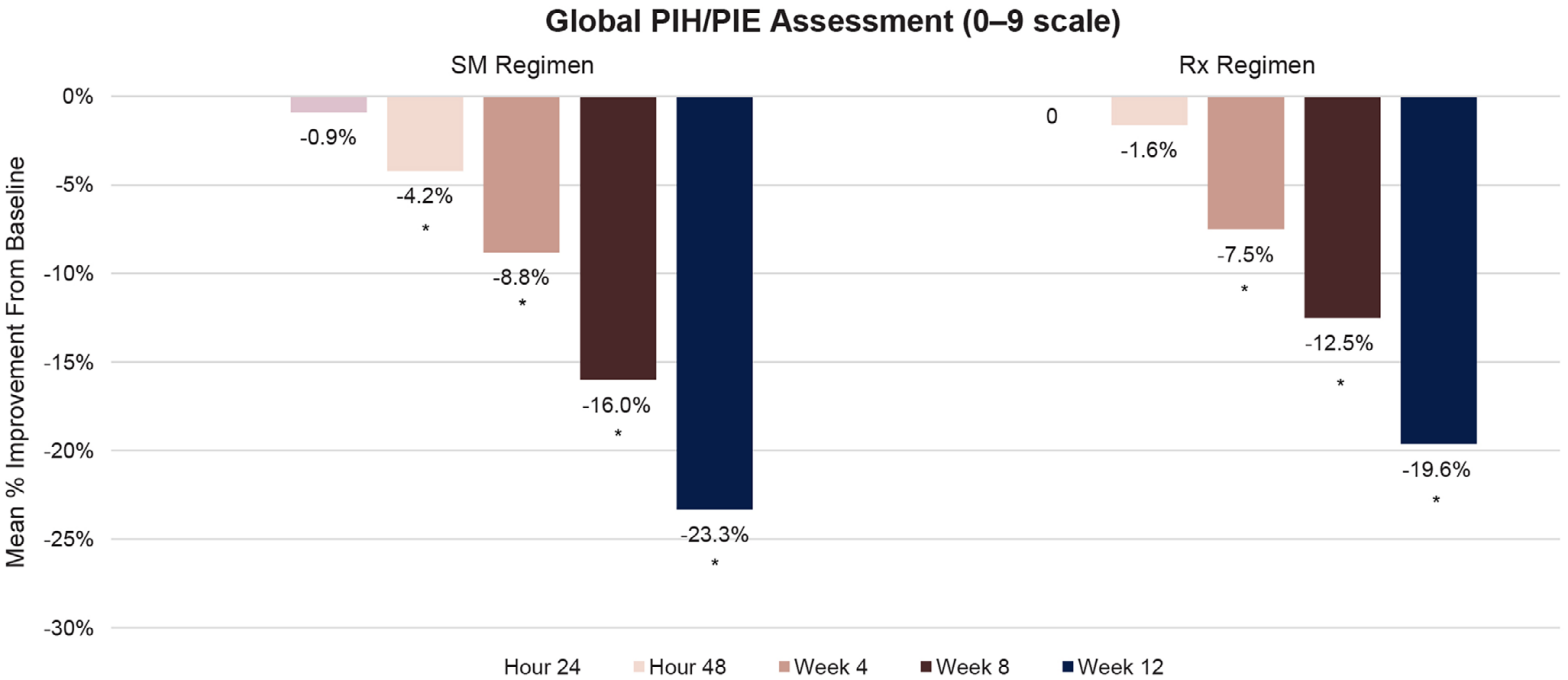
Percentage change from baseline in mean assessment of global PIH/PIE. No statistical significance was observed between treatment groups. PIH/PIE, postinflammatory hyperpigmentation/postinflammatory erythema. *p ≤ 0.004 versus baseline.

Statistically significant improvements versus baseline in global PIH/PIE assessment scores were observed with the SM Regimen starting at 48 h and with the Rx Regimen starting at 4 weeks, with significant improvements observed up to 12 weeks (*p* ≤ 0.004) (Figure 3). The size and darkness of the target PIH/PIE lesions were significantly improved versus baseline at 4, 8, and 12 weeks with both regimens (*p* ≤ 0.002; Table 2). No statistically significant differences were observed between groups in global or target PIH/PIE lesion scores.

#### 3.2.5 Tactile Oiliness

**FIGURE 4:**
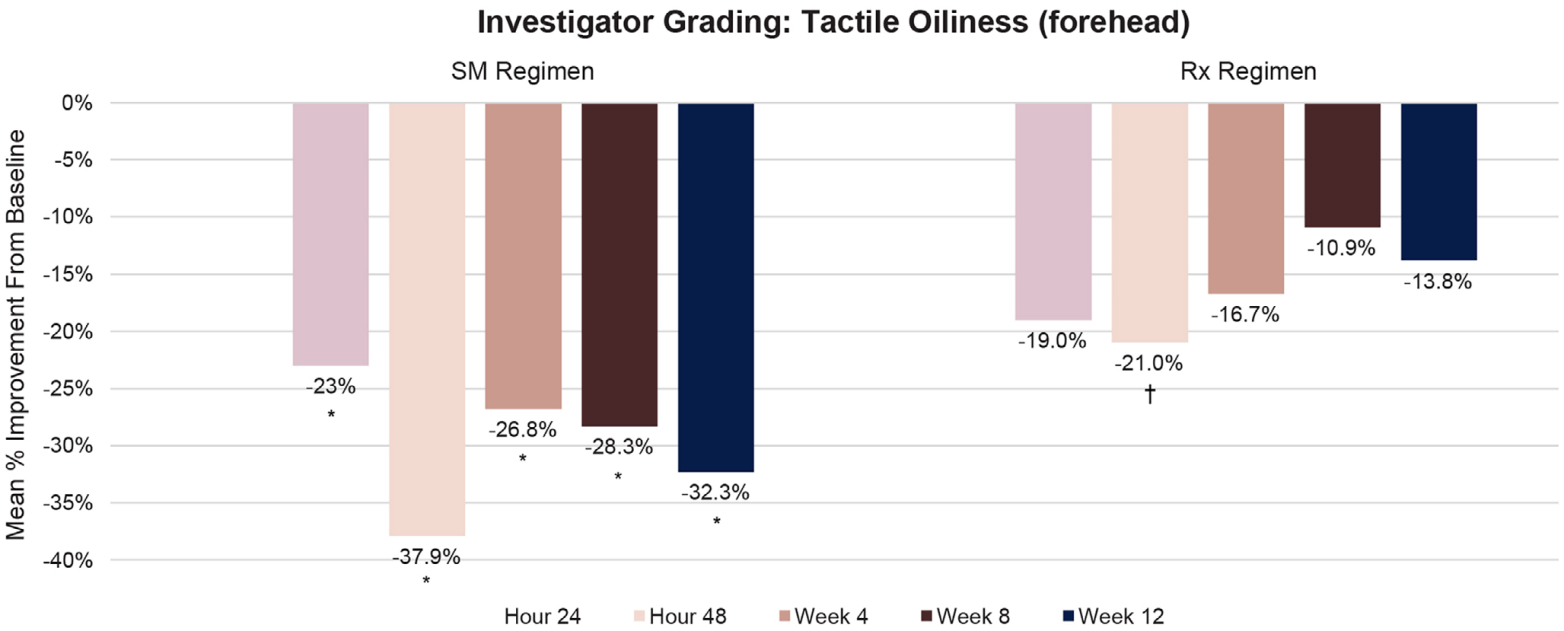
Percentage change from baseline in investigator grading of mean tactile oiliness (forehead) over time. No statistical significance was observed between treatment groups. *p ≤ 0.004 versus baseline; ^†^ *p =* 0.047 versus baseline.

The SM Regimen significantly reduced tactile oiliness at all timepoints compared with baseline (*p* ≤ 0.004), whereas the Rx Regimen showed significant improvements versus baseline at 48 h only (*p* = 0.047; Figure 4). There was no statistically significant difference in tactile oiliness between groups.

### 3.3 Subject Self‐Assessment Questionnaire

Subjects consistently rated the SM Regimen highly across several parameters at 24 and 48 h and at 12 weeks. The highest‐rated parameters included statements about not drying out the skin (≥ 95% of subjects agreed at 24 h), made my skin look and feel less oily (93% at 4 weeks), and made my skin look clearer (90% and 93% of subjects agreed at 48 h and 4 weeks, respectively). Subjects reported favorable treatment satisfaction at all time points for the SM Regimen (100% satisfaction at 24 and 48 h and at 4 and 8 weeks, and 96.4% at 12 weeks).

### 3.4 Photographic Evidence

**FIGURE 5:**
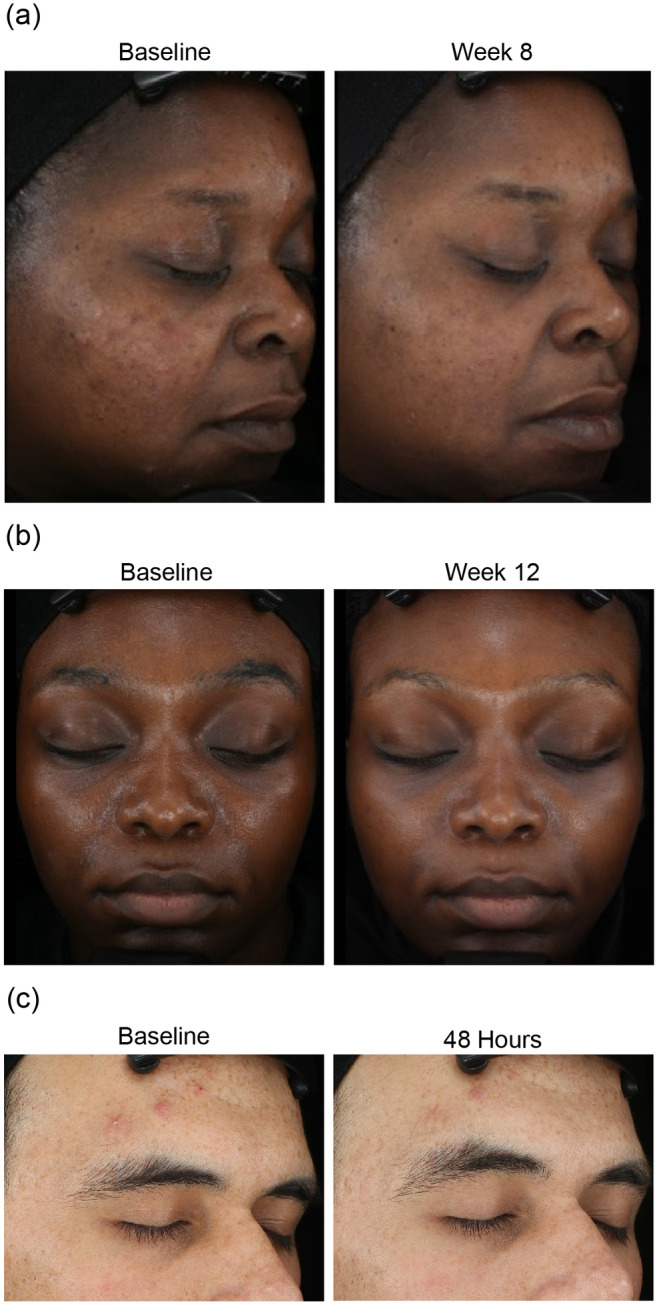
Representative images at baseline and after treatment with SM Regimen. (a) Black/African American female, aged 40 years with FST V, showing a reduction in acne lesions and skin oiliness. (b) Black/African American female, aged 28 years with FST VI, showing reduced skin oiliness and more even skin tone. (c) White/Caucasian male, aged 31 years with FST III, showing rapid reduction in size of inflammatory acne lesions on the forehead. FST, Fitzpatrick skin type.

Representative photographs of improvements from baseline in the appearance of acne and PIH/PIE lesions with the SM Regimen are presented in Figure 5.

### 3.5 Safety and Tolerability

Treatment‐related adverse events were reported in three subjects. One subject in the SM Regimen group experienced a rash on the face that was moderate in severity and possibly treatment related. In the Rx group, one subject developed moderate skin exfoliation and burning sensation on the face, and one subject experienced moderate skin exfoliation and mild pruritus in the upper eyelids and mouth. All events were resolved.

**FIGURE 6:**
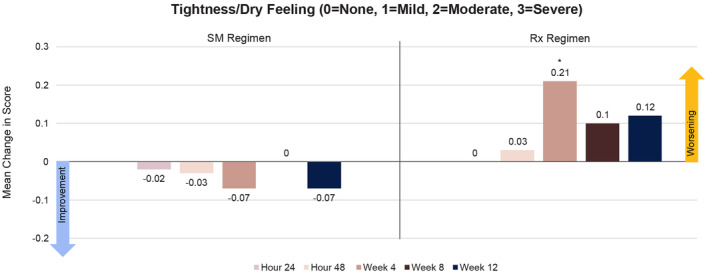
Change from baseline in mean tolerability assessments of tightness and dry feeling over time. No statistical significance compared with baseline at any time point. *Statistically significantly worse versus SM Regimen (*p* = 0.008).

Both treatment regimens were well tolerated at all time points, with mean scores remaining below mild for all tolerability parameters and no significant increases from baseline throughout the study. However, subjects consistently experienced worsened tightness/dry feeling in the Rx Regimen group at follow‐up visits, whereas the SM Regimen group experienced a decrease in tightness/dry feeling. At week 4, subjects in the Rx Regimen experienced significantly worse tightness/dry feeling compared with the SM Regimen group (*p* = 0.008; Figure 6).

## 4 Discussion

Acne can have a substantial aesthetic impact, creating a psychosocial burden on affected individuals regardless of acne severity. The impact of acne on quality of life is greater in individuals who are female, older, or have a longer acne duration (> 5 years) [20], and persons with acne have reported social, psychological, and emotional burdens similar to or greater than those reported by patients with chronic asthma, epilepsy, diabetes, back pain, and arthritis [6]. Adults with greater skin‐related anxiety have also reported a lower intention to participate in sports and exercise, experienced lower self‐esteem, and had worse quality of life [21]. Despite the availability of effective nonprescription and prescription‐grade treatments for mild to moderate adult acne, topical treatments are often limited by tolerability issues, including dryness, peeling, and skin irritation [11]. These issues, related to disruption of the stratum corneum permeability barrier, can result in increased skin sensitivity [22]. Thus, patients may continue to experience distress even if acne has improved with treatment. Considering these issues, there is a need for effective treatments that are gentle on the skin and address tolerability concerns.

In this randomized, double‐blind study of adults with acne, including subjects with PIH/PIE, the SM Regimen (1% salicylic acid cosmetic Acne Clarifying Cleanser and 2% salicylic acid over‐the‐counter Acne Treatment Lotion) was well tolerated and nondrying, and demonstrated significant improvements from baseline in acne severity assessments, PIH/PIE, and tactile oiliness. When compared with a popular Rx Regimen (adapalene 0.1% and BP 2.5%), the SM Regimen showed comparable overall efficacy. However, reductions in total acne lesion counts and noninflammatory lesion counts at 24 h were significantly greater with the SM regimen. The SM Regimen also resulted in significant improvements from baseline in tactile oiliness at all time points, whereas the Rx Regimen significantly improved tactile oiliness only at 48 h.

The SM Regimen demonstrated significant improvements from baseline in global assessments of PIH and PIE as early as 48 h, which were sustained up to 12 weeks. PIH and PIE occur when acne inflammation results in overproduction of melanin or capillary damage, respectively [23]. Although PIE is more common in lighter skin (FST I–III), PIH can occur with any skin type but may be more prevalent and prominent in darker skin [10, 24]. Skin irritation associated with acne treatment may worsen PIH in patients with skin of color or contribute to erythema [23]. The current findings suggest that the SM Regimen, which can be used alone or in combination with a spot treatment [25], may help address the need for gentle and effective acne treatments that reduce PIH and PIE.

Most subjects (> 96%) reported high treatment satisfaction and favorable tolerability with the SM Regimen, including in parameters associated with skin dryness. Current acne treatment formulations can have adverse events, such as dryness and skin irritation, that limit tolerability and treatment adherence [11]. In addition, adults often have additional needs related to skin aging, such as reduced barrier function, impaired hydration, and changes in skin quality (e.g., larger pores; wrinkles; dull, blotchy, and rough skin; hyperpigmentation; erythema; and dryness) [26].

The SM Regimen takes a multifactorial approach to acne control, considering the needs of adult skin. Specifically, ingredients in the SM Regimen, including bakuchiol, niacinamide, and a synergistic complex of chaulmoogra, black cumin, manuka, and magnolia, help address acne without drying the skin and work to restore and maintain a good balance of sebum. Overabundance of sebum can promote acne‐related inflammation, as oxidation of lipids in sebum triggers the release of inflammatory mediators in the skin [27]. An imbalance of oxidants and antioxidants in acne‐prone skin contributes to this process [27]. Ingredients with antioxidant properties, such as bakuchiol and niacinamide, were included in the SM Regimen with the aim of restoring this balance, preventing sebum oxidation, and reducing inflammation.

Strengths of this study include that the SM Regimen was compared with the gold standard prescription combination topical treatment and was evaluated in a diverse population of subjects, and the study met target enrollment for subjects with PIH/PIE and with darker skin types. Limitations include that this was a single‐center study and the lack of an adolescent cohort. It is possible that observed benefits of the SM Regimen could extend to adolescents because the etiology of acne may be similar in adults and adolescents. Additional research is warranted in larger, multi‐site studies to evaluate the effectiveness and tolerability of the SM Regimen in adolescents with acne. Also, the small sample size could be a potential study limitation; however, the number of patients enrolled in this study aligns with other studies investigating safety and efficacy of topical acne products [12].

In this randomized study of adults with acne, the SM Regimen effectively reduced acne severity and skin oiliness while evening out skin tone without overdrying or irritating the skin. Most subjects (> 96%) reported high satisfaction with the SM Regimen at all time points, suggesting it may help address the need for an effective acne treatment that respects the needs of adult skin.

## Author Contributions

Study design: Priscilla Huang, Elizabeth T. Makino, and Rahul C. Mehta. Principal investigator: Elizabeth T. Makino. Study investigator: Cecilia L. Pak. Enrolled patients: Cecilia L. Pak. Collection and assembly of data: Cecilia L. Pak. Data analysis: Cecilia L. Pak. Data interpretation: All authors. Manuscript review and revisions: All authors. Final approval of manuscript: All authors.

## Ethics Statement

The authors confirm that the ethical policies of the journal, as noted on the journal's author guidelines page, have been adhered to and the appropriate ethical review committee approval has been received. The study was approved by an institutional review board (Advarra IRB, Columbia, MD, USA) and conducted in accordance with all applicable guidelines for the protection of human subjects for research as required by 21 Code of Federal Regulations (CFR) 50.25 and the accepted standards for Good Clinical Practice.

## Consent

All patients have provided written informed consent for their photos to be published.

## Conflicts of Interest

Priscilla Huang: employee of Allergan Aesthetics, an AbbVie Company, and may own AbbVie stock. Olivia Supan: employee of Allergan Aesthetics, an AbbVie Company, and may own AbbVie stock. Cecilia L. Pak: employee of SGS Stephens Inc. Rahul C. Mehta: former employee of Allergan Aesthetics, an AbbVie Company, and may own AbbVie stock. Elizabeth T. Makino: employee of Allergan Aesthetics, an AbbVie Company, and may own AbbVie stock.

## Data Availability

The data that support the findings of this study are available from the corresponding author upon reasonable request. Data sharing: AbbVie is committed to responsible data sharing regarding the clinical trials we sponsor. This includes access to anonymized, individual, and trial‐level data (analysis data sets), as well as other information (e.g., protocols, clinical study reports, or analysis plans), as long as the trials are not part of an ongoing or planned regulatory submission. This includes requests for clinical trial data for unlicensed products and indications. These clinical trial data can be requested by any qualified researchers who engage in rigorous, independent, scientific research, and will be provided following review and approval of a research proposal, Statistical Analysis Plan (SAP), and execution of a Data Sharing Agreement (DSA). Data requests can be submitted at any time after approval in the United States and Europe and after acceptance of this manuscript for publication. The data will be accessible for 12 months, with possible extensions considered. For more information on the process or to submit a request, visit the following link: https://vivli.org/ourmember/abbvie/ then select “Home.”
